# Dendrobine Ameliorates Alzheimer’s Disease-like Pathology and Cognitive Decline in 3 × Tg-AD Mice

**DOI:** 10.3390/brainsci14030231

**Published:** 2024-02-28

**Authors:** Wei Zhang, Juan Huang, Jingshan Shi

**Affiliations:** Key Laboratory of Basic Pharmacology of Ministry of Education and Joint International Research Laboratory of Ethnomedicine of Ministry of Education, Zunyi Medical University, Zunyi 563003, China; zhangweizwszbd@163.com (W.Z.); huangjuanzmu@163.com (J.H.)

**Keywords:** Dendrobine, spatial memory, synaptic loss, β-amyloid, tau

## Abstract

Previous studies have shown that *Dendrobium nobile* Lindl. alkaloids (DNLAs) have neuroprotective effects in several Alzheimer’s disease (AD) models. Dendrobine (DDB) is one of the monomer components with the highest content in DNLAs. However, the effects of DDB on cognitive impairments in AD remain unknown. In this study, we investigated the efficacy of DDB in 3 × Tg-AD mice to determine whether DDB was a key component of the anti-AD effect of DNLAs. Five-month mice were intragastrically administrated with DDB (10 and 20 mg/kg/d) or DNLAs (20 mg/kg/d) for seven consecutive months, and the effects of DDB and DNLAs were evaluated at twelve months. The results revealed that 3 × Tg-AD mice treated with DDB showed enhanced nesting ability. DDB also effectively rescued spatial learning and memory deficits in 3 × Tg-AD mice. Meanwhile, DDB treatment prevented the loss of dendritic spine density, with increased expression levels of synaptophysin, PSD95, and NCAM in the hippocampus. Finally, DDB ameliorated the increase in APP, sAPPβ, CTF-β, and β-amyloid peptides, accompanied by the promotion of GSK phosphorylation at the Ser9 site, thereby reducing hyperphosphorylated tau levels. As the active component of DNLA, DDB can preserve cognitive function, alleviate neuronal and synaptic defects, and improve APP/tau pathology in 3 × Tg-AD mice.

## 1. Introduction

Alzheimer’s disease (AD), the most common cause of dementia in the elderly, is now becoming a primary public health concern [1]. Clinically, with the development of the disease, patients with AD show a progressive decline in memory and deterioration of cognitive and motor functions [2,3]. The main pathological hallmarks of AD are extracellular senile plaques composed of β-amyloid peptides (Aβs) and intracellular neurofibrillary tangles (NFTs) composed of abnormally phosphorylated tau protein in the associative cortex and hippocampus, which are key structures in learning and memory [4]. Other changes include neuronal loss and synaptic dysfunction [5]. Aβ interacts with phosphorylated tau and disturbs neuronal, especially synaptic, structure and function, leading to cognitive decline in AD patients [6]. Due to the multifactorial pathology of AD, developing effective therapies is challenging. Currently approved drugs can only attenuate symptoms but are unable to reverse the AD process. Thus, there is a huge unmet need for drugs that truly prevent or treat the disease.

**Figure 1:**
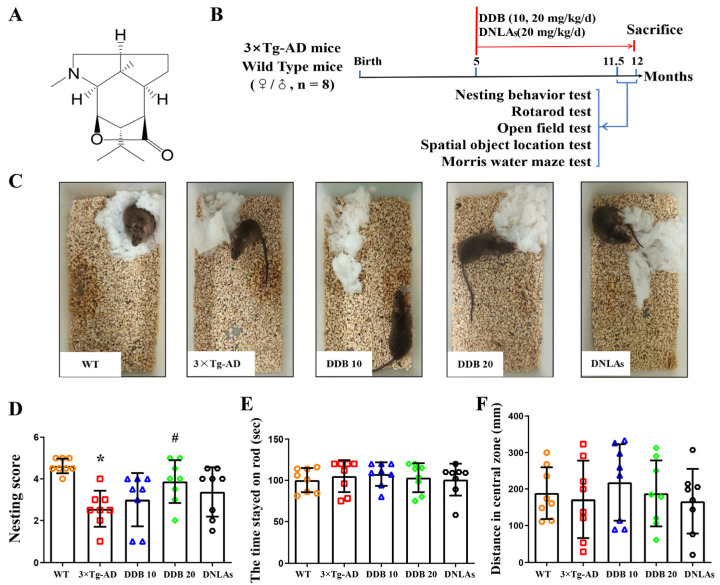
Effects of DDB on behavioral indices in 3 × Tg-AD mice: (**A**) chemical structure of Dendrobine; (**B**) experimental schedule of drug administration and behavior studies, the red arrow indicates the phase of drug administration. (**C**) nesting behavior test. Representative pictures of cotton nest construction in WT, 3 × Tg-AD mice, and 3 × Tg-AD mice treated with DDB or DNLAs. (**D**) rotarod test. Quantification of residence time on rob. (**E**) bar graph showing the results in mice from different groups. (**F**) open-field test. Quantification of distance traveled in the central zone. Each colored symbol representing a different individual mouse is colored according to the corresponding mouse group. n = 8 mice per group. Error bars represent the mean ± SD. * *p* < 0.05 versus WT; # *p* < 0.05 versus 3 × Tg-AD.

*Dendrobium nobile*, a precious epiphytic orchid, has a long history of use as a medicinally important herb and health food in China [7]. It contains a variety of active ingredients, such as alkaloids and polysaccharides. With a wide range of antiaging, antioxidant, immunomodulatory, hypoglycemic, and hypolipidemic activities, these ingredients exert therapeutic effects by alleviating tumors, diabetes, hyperlipidemia, and aging-associated neurodegenerative diseases [8,9]. *Dendrobium nobile* Lindl. alkaloids (DNLAs) extracted from *Dendrobium nobile* contain a variety of monomer components, including Dendrobine (DDB, 92.6%) (Figure 1A), Dendrobine-N-oxide (3.3%), Nobilonine (2.0%), Dendroxine (0.9%), 6-Hydroxynobilonine (0.32%), and 13-Hydroxy-14-oxodendrobine (0.07%), etc. [10]. Our previous studies have demonstrated that DNLAs have neuroprotective activity in both in vivo and in vitro models of AD. For instance, DNLAs, as autophagy inducers, can promote lysosomal acidification and the clearance of toxic protein aggregates in APP/PS1 [11] and SAMP8 mice [12], increase the expression of neurotrophic factors in Aβ_25–35_-treated mice [10] and decrease neuronal apoptosis and the hyperphosphorylation of tau proteins [13]. DNLAs also reverse memory impairment induced by lipopolysaccharide in rats [14]. Here, we were interested in whether DDB, as the main monomer component with the highest content in DNLAs, has preventive effects on AD progression and its underlying mechanisms.

In the present study, we evaluated the efficacy of DNLAs extract DDB on improving cognitive function, neuronal and synaptic efficacy, and the degradation of APP/tau products in the triple-transgenic mouse model of Alzheimer’s disease (3 × Tg-AD), which expresses mutant human APP, presenilin, and tau proteins. Our data demonstrate that DDB is the active ingredient of DNLAs. This is reflected in the effects of DDB on the remission of synaptic loss, as well as improvement in cognitive and memory impairments. The mechanisms may be related to the inhibition of the β-secretase processing of APP and Aβ production, accompanied by the amelioration of tau hyperphosphorylation by restoring the glycogen synthase kinase-3β (Gsk3β) phosphorylation at the Ser9 site in the hippocampus of 3 × Tg-AD mice.

## 2. Materials and Methods

### 2.1. Experimental Animals

To conduct the experiments, 3 × Tg-AD [B6;129-Tg (APPSwe, tauP301L) 1Lfa Psen1 tm1Mpm/Mmjax] mice were purchased from Jackson Laboratories (Bar Harbor, ME, USA, stock no. 34830-JAX). They were bred and maintained at the Key Laboratory of Basic Pharmacology of Zunyi Medical University (Certificate no.: SYXK 2021-0003). All mice were raised in group-housed conditions and provided with free intake of water and food in standard plastic cages with chip shavings for bedding material. Additionally, 3 × Tg-AD mice were inbred to produce offspring, which were genotyped to select PS1/M146V, APPSwe, and tauP301L familial AD gene mutations mice. The animals were kept in a well-ventilated room at room temperature (22 ± 2 °C), humidity (50–60%), and a 12/12 h light–dark cycle. Age-matched nontransgenic litter-mate WT (wild-type) mice were used as controls. Mice of 5 months of age were used. All experiments and animal care conformed to the guidelines of the National Institutes of Health and were approved by the Laboratory Animal Welfare and the Ethics Committee of Zunyi Medical University (permit number: ZMU11-2203-468).

### 2.2. Drug Treatment

Briefly, five-month-old 3 × Tg-AD mice (male and female) were randomly assigned to 3 × Tg-AD, DDB, and DNLA treatment groups (n = 8), which were intragastrically administered with DDB (10, 20 mg/kg/d) or DNLAs (20 mg/kg/d) daily for seven consecutive months. DDB and DNLAs were dissolved in distilled water containing 0.5% tween 80 at a final concentration of DDB 1, 2 mg/mL, DNLAs 2 mg/mL. Five-month-old WT mice were treated with vehicle (0.5% tween 80) by oral gavage for seven months. The groups administered with 20 mg/kg/d and 10 mg/kg/d of DDB will be referred to as DDB 20 and DDB 10, respectively.

### 2.3. Behavioral Tests

#### 2.3.1. Nesting Behavior Test

Nesting behavior was investigated as an assay for social behavior in this study. The mice were housed in individual home cages to adapt a solitary environment for 48 h prior to the test. All enrichment objects were removed from the home cages of the mice and replaced with corn cob bedding. Two hours prior to the onset of the dark phase of the circadian cycle, a 5 cm square was supplied to each mouse in its home cage as nesting material, and eighteen hours were allowed to pass, after which the nest construction was scored along the 5 point scaling system of Deacon [15]. The identification cards were coded to render the experimenters blind to the genotype and experimental treatments of each subject. The final nesting score for each mouse was the mean of the scores from 2 experimenters.

#### 2.3.2. Rotarod Test

The rotarod test was used to evaluate the motor performance of mice [16]. A rotarod apparatus with automatic timers and falling sensors (Chengdu Techman Software Co., Ltd., Chengdu, China, ZB-200) was used. On the day preceding the test, all mice were trained three times to stay on a rotating rod at 5 rpm, 10 rpm, and 15 rpm for 100 s each time, during which the animal was placed back on the rolling rotarod immediately after falling. In the test phase, the mice were placed on the rod at an initial rotation rate of 5 rpm, and then steadily increased by 5 rpm every 30 s. The latency to falling over a 120 s period was recorded. The apparatus was cleaned with 75% ethanol between each trial.

#### 2.3.3. Open Field Test

Locomotor activity was assessed by an open field test [12]. Mice were individually tested in an open field arena (consisting of a 40 cm × 40 cm × 40 cm box) that included a central area taking up 50% of the bottom area. Free locomotion was tracked for 5 min with a video-tracking camera system (Topscan, CleverSys, Inc., Reston, VA, USA), and the move distances in the central area were automatically recorded. After each period, the arena was wiped with 75% ethanol.

#### 2.3.4. Morris Water Maze

Spatial learning and memory performance of mice were detected with a Morris water maze. It was carried out as previously described [17]. Briefly, the water maze pool (120 cm in diameter) made of white polyvinyl chloride (PVC) was filled with water (24 ± 2 °C). An escape platform (10 cm in diameter) was placed into the pool and hidden 1.5 cm below the water surface. During the training period, mice went through 3 trials per day for 6 consecutive days. Here, mice were allowed to freely swim for 60 s to search for the hidden platform. The trajectory taken by each mouse was monitored by a video-tracking camera system (Topscan), and the escape latency time to find the platform was recorded during each trial. If the mouse found the platform within the 60 s allowed, it was permitted to remain on the platform for 20 s. If the mouse failed to find the platform within the maximum time limit, it was manually guided to the platform for a 20 s period. Probe trials were carried out 1 or 24 h after the last training session in which the platform was removed and the mice were allowed to explore for 60 s. The parameters measured during the probe trial included the time spent in the target quadrant, swimming speed, and the number of crossings through the platform location.

#### 2.3.5. Spatial Object Location Test

Mice first familiarize the two objects placed in two corners of the open field arena for 5 min. The position of the object in the arena was kept unchanged for 24 h after the familiarization sessions, and the position of the object was shifted from one corner to the opposite corner. During the retention period, mice re-entered the arenas where they were granted 5 min to explore both objects. The discrimination index was calculated by the time that mice spent exploring the displaced object–the time that mice spent exploring the nondisplaced objects/the total exploration time. After each period the arena and objects were cleaned with 75% ethanol.

### 2.4. Nissl Staining

After behavioral testing, the mice were anesthetized by intraperitoneal injection of sodium pentobarbital (100 mg/kg) and perfused intracardially with cold saline. All mice were then decapitated, and the brains were immediately removed and fixed in 4% paraformaldehyde for 48 h [12]. The fixed brains were dehydrated, embedded in paraffin and continuously sliced along the coronal plane into 4 μm thick sections. Then, brain slices were stained using a toluidine blue solution for 20 min. Finally, the severity of the neural injury was evaluated by counting the number of surviving neurons in the hippocampal CA1 areas.

### 2.5. Golgi Staining

The mice were decapitated under deep anesthesia, and brains were removed from the skull and washed with normal saline. Then, Golgi staining was performed according to the previous report [18]. In brief, the brains were incubated in potassium dichromate with chloral hydrate mixed staining solution and moved to a 1% silver nitrate solution. The morphology of neuronal dendrites and dendritic spines in the hippocampal DG area was detected, and the spine density of the dendrites of about 10 min length was counted using Image J software (x64)1.8.0.

### 2.6. Immunohistochemistry (IHC) Staining

For IHC staining [11], deparaffinized and rehydrated brain sections were treated for 20 min with 0.3% hydrogen peroxide (H_2_O_2_) to quench endogenous peroxidase activity, washed with PBS, exposed to goat serum for 30 min at room temperature, and incubated overnight at 4 °C with anti-beta amyloid (MOAB-2, NBP2-13075SS, Novus, Abingdon, UK). Biotinylated secondary antibodies and avidin-peroxidase complex were applied after primary antibodies. Slides were then incubated with 3,3′-diaminobenzidine (DAB Kit, DA1010, Solarbio, Beijing, China) and then mounted with neutral balsam. Images were observed under the microscope (Nikon, Tokyo, Japan).

### 2.7. Immunofluorescent Staining

Mouse brain slices were incubated with 3% goat serum for 30 min at 37 °C to block nonspecific antibodies sites followed by incubation with primary antibodies overnight at 4 °C [18]. The primary antibodies used in this study were anti-MAP2 (17490-1-AP, Proteintech, Rosemont, IL, USA) and anti-synaptophysin (ab32127, Abcam, Cambridge, UK). After washing with PBS, Alexa Fluor conjugated secondary antibodies incubation was performed for 2 h at room temperature. Then, 4′,6-diamidine-2′-phenylindole dihydrochloride (DAPI, C1005, Beyotime, Nantong, China) was used as a nuclear counterstain. All the images of the hippocampal region were captured under the confocal microscope (Olympus, Tokyo, Japan).

### 2.8. Western Blotting

For Western blotting, hippocampal tissues were harvested, homogenated and boiled at 100 °C for 10 min in the loading buffer (P0015-L, Beyotime, Haimen, China). Lysates (30 μg of protein) were fractionated by SDS-polyacrylamide gel electrophoresis on a 10% gradient gel and then the separated proteins were transferred to nitrocellulose membranes (IPVH00010, Millipore, Darmstadt, Germany). The membranes were then blocked with 5% nonfat milk dissolved in TBST for 1 h, and then exposed to the following primary antibodies at 4 °C overnight: anti-β-site APP cleaving enzyme 1 (BACE1, Abcam, ab108394), anti-γ-secretase (PSEN1, Abcam, ab76083), anti-β-amyloid precursor protein (APP, Abcam, ab32136), anti-GSK3β (22104-1-AP, Proteintech, Rosemont, IL, USA), anti-p-ser9-GSK3β (Abcam, ab107166), anti-Human-sAPPβ (6A1, 10321, IBL, Minneapolis, MN, USA), anti-CTF antibody (A8717, Sigma, St. Louis, MO, USA), anti-Phospho-tau (Ser202, Thr205; AT8, MN1020, Invitrogen, Carlsbad, CA, USA), anti-tau-5 (Invitrogen, MA5-12808), anti-tau (Phospho-Ser396) (SAB, #11102), anti-PSD95 (Abcam, ab18258), anti-tau (Phospho-Ser404, SAB, #11112, ThermoFisher, Waltham, MA, USA), anti-NCAM (Abcam, ab95153), anti-Synaptophysin (Abcam, ab32127), anti-GAPDH (Proteintech, 10494-1-AP), and anti-β-actin (Proteintech, 20536-1-AP). The immune complexes were detected with horseradish peroxidase-conjugated secondary antibodies (Proteintech, SA00001-1, -2) at 37 °C for 1 h and enhanced chemiluminescence reagents. The blots were densitometric analyzed by Quantity One software (ImageLab 5.2, Bio-Rad, Hercules, CA, USA).

### 2.9. Statistical Analysis

The data are presented as mean ± standard deviation (SD) and analyzed using GraphPad Prism (San Diego, CA USA) statistical software (Version 8.3.0). The one-way analysis of variance procedure followed by Dunnett’s multiple comparison test was used to determine the differences among groups. A two-way analysis of variance among the performance record in the Morris water maze test was used and a *p* value of <0.05 was considered statistically significant.

## 3. Results

### 3.1. DDB Ameliorate Cognitive Decline and Memory Impairment in 3 × Tg-AD Mice

Normal sensory and locomotor functions were found in 3 × Tg-AD mice before 6 months, and a decline in learning and memory was found afterward [19]. To evaluate the effects of DNLA extract and DDB on cognition and locomotor function in 3 × Tg-AD mice, a series of behavioral tests were sequentially performed (Figure 1B). During the nesting behavior test, compared with the WT mice, 3 × Tg-AD mice exhibited a clear decrease in nesting behavior, and they left the cotton material mostly undisturbed. However, mice in the DDB 20 group chewed and tore the cotton pieces and arranged them into a well-defined nest, indicating that DDB administration can mitigate the decline in nesting behavior (Figure 1C,D). Next, the muscular coordination and balance of mice were determined by the rotarod test, and five groups of animals exhibited similar residence time on the rod (Figure 1E). An open field test was used to detect locomotor exploratory activity of the mice; there were no statistically significant differences in exploring the central area of the open field arena among the five groups. These observations indicate that compared to wild-type mice, 3 × Tg-AD mice at 12 months of age, or DDB administration, did not elicit any difference in the motor ability and locomotor activity of these mice (Figure 1F).

**Figure 2:**
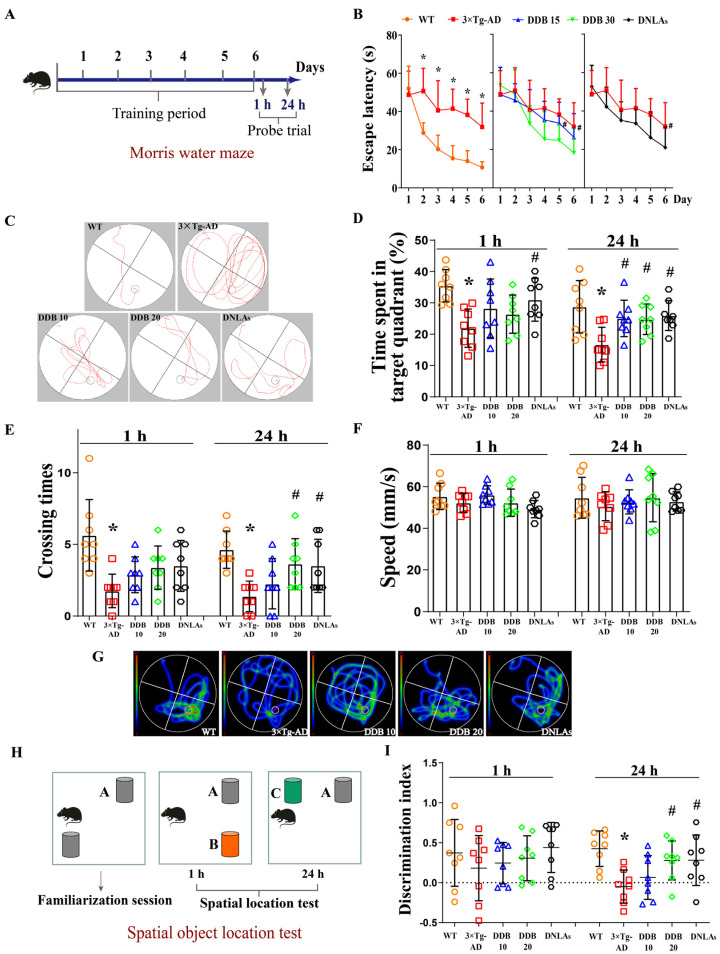
DDB rescues long-term spatial memory deficit in 3 × Tg-AD mice. (**A**) Timeline of the Morris water maze test. (**B**) Quantification of latency time taken to reach the hidden platform. (**C**) Representative searching trace on day 6 of the training. (**D**) The percentage of time spent in the target quadrant during the probe trials. (**E**) The number of times of crossing the position of the platform. (**F**) Swimming speed during the probe trial. (**G**) Representative moving patterns of mice in each group in the probe trials. (**H**) The experimental design of spatial object location test. The test conducted 1 and 24 h after the familiarization sessions. (**I**) The discrimination index of 1 and 24 h test trial. Each colored symbol representing a different individual mouse is colored according to the corresponding mouse group. * *p* < 0.05 versus object A or WT, # *p* < 0.05 versus 3 × Tg-AD. n = 8 mice per group. Data are mean ± SD.

Furthermore, we employed the Morris water maze to evaluate the spatial memory ability of the mice (Figure 2A). After 6 consecutive days of platform learning trials, the 3 × Tg-AD mice exhibited a clear longer time to locate the hidden platform compared with the WT mice. Importantly, the 3 × Tg-AD mice treated with DDB or DNLAs had a significantly shorter escape latency time than the vehicle-treated 3 × Tg-AD mice by days 5 and 6, showing that impaired learning ability and reference spatial memory was alleviated (Figure 2B,C). In the probe trial at 1 h, mice in the five groups showed comparable time spent in the target quadrant, crossing number on the platform and swimming speed, indicating that DDB or DNLAs have no effect on motion function and short-term memory. In the probe trial at 24 h, the treatment of DDB or DNLAs displayed an improvement in spatial learning and memory as demonstrated by increased crossing times of the platform and swimming time in the target quadrant compared to the 3 × Tg-AD mice, even though there was no change in swimming speed (Figure 2D–G). Collectively, these results indicate that DDB ameliorated long-term spatial memory deficits without increasing swim speed, suggesting that this intervention did not simply improve motor function. This recuperation of memory ability by DDB treatment was also evident from the results of the spatial object location test (Figure 2H), on which discrimination index in the 24 h but not 1 h test trials session was significantly decreased in 3 × Tg-AD mice compared with WT mice, but 7-month DDB treatment increased the discrimination index in 3 × Tg-AD mice (Figure 2I). These results suggest that DNLAs and DDB improved long-term memory impairment in 3 × Tg-AD mice.

To verify whether the DDB-mediated improvement in cognitive function of 3 × Tg-AD mice can be attributed to its effect to attenuate APP and tau pathology, the levels of different forms of APP- and tau-related protein in the hippocampus and cortex of mice were determined by Western blot.

### 3.2. DDB Degraded APP Amyloidogenic Processing in 3 × Tg-AD Mice

**Figure 3:**
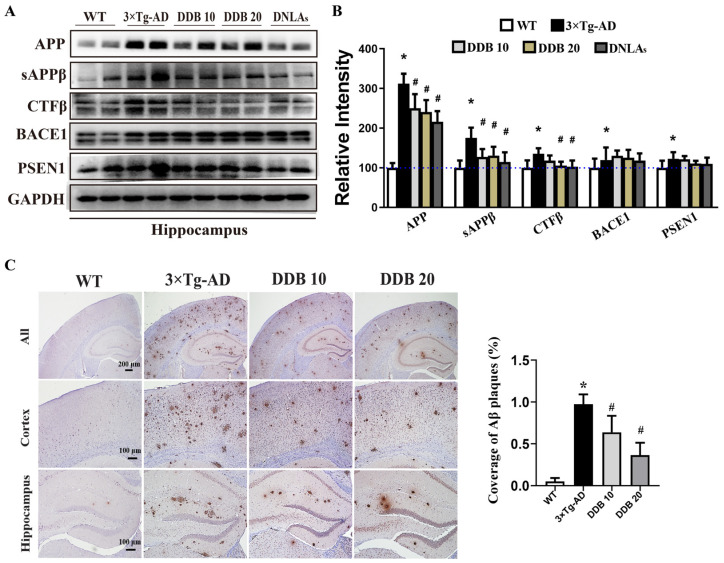
DDB treatments decrease APP pathway-related proteins and Aβ overproduction in 3 × Tg-AD mice. (**A**) Western blots for APP, sAPPβ, CTFβ, PSEN1 and BACE1 in mouse hippocampus. (**B**) Quantification of the relative protein expression levels after normalization to the GAPDH in each sample. (**C**) Representative images and quantitative analysis for the Aβ deposition (MOAB-2 staining) in mouse brain. n = 3–4 mice per group. * *p* < 0.05 versus WT, # *p* < 0.05 versus 3 × Tg-AD. Data are mean ± SD.

Figure 3A,B show that the expression of APP protein in 3 × Tg-AD mice was significantly higher than that of age-matched WT mice. In contrast, DDB or DNLAs treatment reduced the expression of APP protein, along with the decrease of APP metabolites sAPPβ and CTFβ, but did not affect the expression of APP cleavage enzymes PSEN1 and BACE1 in 3 × Tg-AD mice.

To further determine whether DDB-elicited suppression of APP amyloidogenic processing improves Aβ burden in 3 × Tg-AD mice brains, we performed immunohistochemistry staining to observe the Aβ peptide load by MOAB-2 antibody (Figure 3C), which recognizes the unaggregated, oligomeric and fibrillar form of Aβ. By quantitative analyses, the Aβ deposits were significantly increased in the hippocampus and the cortex regions of 3 × Tg-AD mice compared with WT mice and were restored after DDB treatment. Taken together, the result indicated that DDB inhibited the amyloidogenic APP processing, thus causing Aβ secretion decrease.

### 3.3. DDB Attenuates Tau Hyperphosphorylation in 3 × Tg-AD Mice

**Figure 4:**
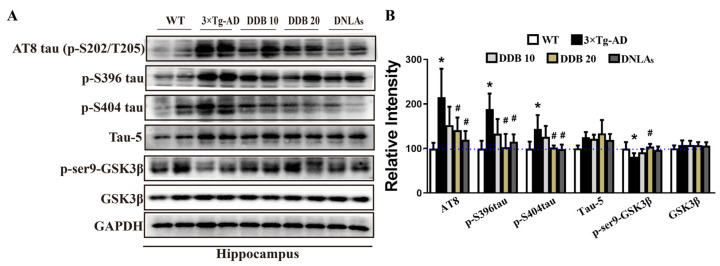
DDB treatments decrease tau phosphorylation levels in 3 × Tg-AD mice. (**A**) Western blots for p-S202/T205(AT8), p-S396, p-S404, total tau (tau-5), p-Ser9-GSK3β and GSK3β in mouse hippocampus and cortex. (**B**) Quantification of the relative p-tau, total tau, GSK3β and p-Ser9-GSK3β expression levels after normalization to the GAPDH or GSK3β.The blue dashed line indicates a relative strength value of 100. n = 4 mice per group. * *p* < 0.05 versus WT, # *p* < 0.05 versus 3 × Tg-AD. Data are mean ± SD.

The effect of DDB on tau and p-tau protein levels is shown in Figure 4; the results indicated that tau phosphorylation level was increased at Ser202/Thr205 (p-S202/p-T205), Ser396 (p-S396), and Ser404 (p-S404) sites in the hippocampal and cortex extracts of the 3 × Tg-AD mice comparing to the WT mice, whereas DDB treatment effectively attenuated increased expression of these p-tau protein levels. There was no significant change in the total tau (tau-5) level.

Regulation of GSK3 activity plays an important role in AD-like tau hyperphosphorylation [20]. To explore the underlying mechanisms that DDB attenuated tau hyperphosphorylation, GSK3β phosphorylation levels at the sites of Ser9 induced by DDB in mice brain were further examined. Figure 4 shows that the protein levels of p-Ser9-GSK3β were decreased in the hippocampus of 3 × Tg AD mice compared with WT mice, which were clearly enhanced with DDB treatment. GSK3β level remained comparable among these groups.

### 3.4. DDB Prevents Synaptic Degeneration in 3 × Tg-AD Mice

**Figure 5:**
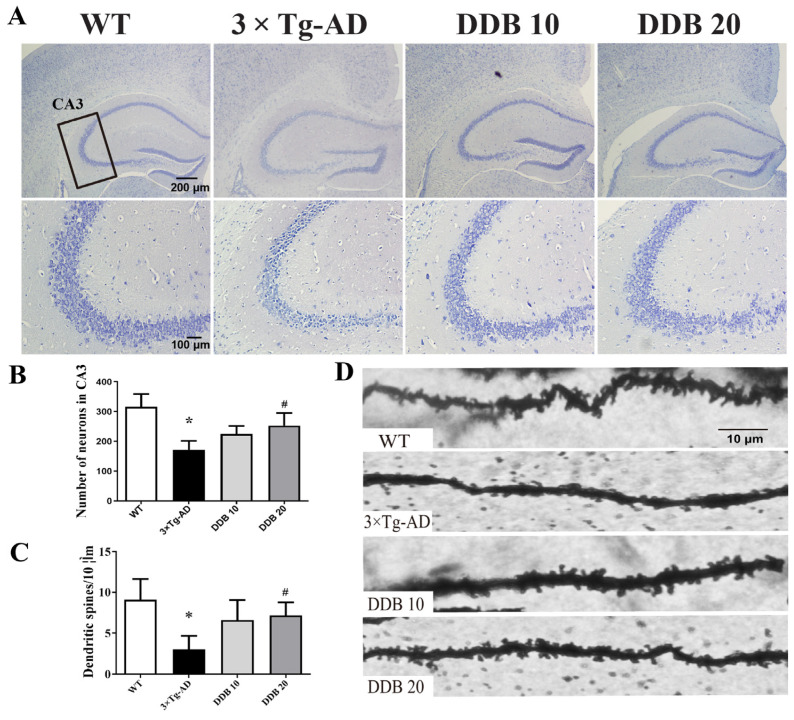
DDB restored neuronal and dendritic spine degeneration in the hippocampus of 3 × Tg-AD mice. (**A**) Nissl staining of the brain slices from WT, 3 × Tg-AD, DDB 10 and DDB 20 mouse. The rectangular area shows the CA3 subregion of the hippocampus. (**B**) Quantitative analysis of the neuron numbers in CA3 regions of the hippocampus. (**C**) Representative micrographs of dendritic spines morphology in dentate gyrus area from Golgi impregnated hippocampus. (**D**) Quantitative analysis of spine number per 10 μm dendrite segment. n = 3–4 mice per group. * *p* < 0.05 versus WT, # *p* < 0.05 versus 3 × Tg-AD. Data are mean ± SD.

To investigate whether DDB inhibits neurodegeneration in mouse brains, we first evaluated the neuron survival by Nissl staining and found that the number of neurons in the hippocampal CA3 region of 3 × Tg-AD mice significantly decreased compared with WT mice, while there were no differences between DDB treatment group and 3 × Tg-AD mice (Figure 5A,B). Next, we observed that synaptic configuration changes by measuring dendritic spine density in the hippocampal dentate gyrus area of mice. As shown in Figure 5C,D, the averaged spine density of 3 × Tg-AD mice revealed in Golgi staining was decreased compared to WT mice; however, DDB effectively reversed the dendritic spine loss.

**Figure 6:**
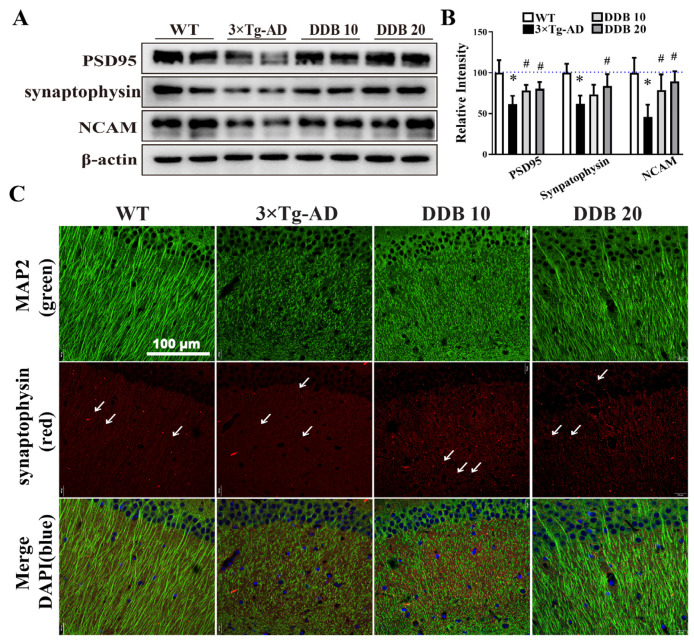
DDB recovered synaptic protein expression in the hippocampus of 3 × Tg-AD mice. (**A**) postsynaptic density 95 (PSD95), synaptophysin and neural cell adhesion molecule (NCAM) expression levels were detected by Western blotting in mouse hippocampus. β-actin was used as a loading control. (**B**) Quantitative analysis of the synaptic protein levels. (**C**) Immunofluorescence localization of MAP2 (green), synaptophysin (red, white arrowheads) and DAPI (blue, nuclei) in the hippocampal CA1 area of 3 × Tg-AD mouse brain slices. n = 4 mice per group. * *p* < 0.05 versus WT, # *p* < 0.05 versus 3 × Tg-AD. Data are mean ± SD.

The protein levels of postsynaptic density 95 (PSD95), synaptophysin and neural cell adhesion molecule (NCAM) were decreased markedly in the hippocampus of the 3 × Tg-AD mice group, while the synaptic degeneration was significantly alleviated through upregulation of the synaptic protein expression in the hippocampal neurons of mice receiving DDB treatment (Figure 6A,B). Consistent with these experiments, immunofluorescence staining also showed that in WT mice, synaptophysin was localized to dense particles arranged along the green MAP2 labeled dendrites, whereas in 3 × Tg-AD mice, loss of synaptophysin staining puncta on MAP2-positive dystrophic dendrites was seen (Figure 6C). In total, 20 mg/kg DDB-treated 3 × Tg-AD mice revealed that synaptophysin and MAP2 distribution resembles that seen in WT mice. These results suggested that DDB prevented neuronal loss and synaptic impairment, which may be correlated to neural efficacy and thus enhance the ability of mice to cognize and memorize.

## 4. Discussion

AD is the most prevalent age-associated neurodegenerative disorder of unknown origin; despite decades of research, there is still no effective pharmacotherapy for this condition. The current “gold standard” strategies are aimed at prevention or mitigation of cognitive decline rather than curative treatments for the underlying disease. *Dendrobium nobile* is a traditional Chinese medicine with homology of medicine and food, which provides a beneficial effect on preventing senescence. Previous studies have shown that DNLAs, as natural alkaloid extracts of *Dendrobium nobile*, have protective effects in mouse models of AD and Parkinson’s disease [12,21]. DDB is a critical component of the DNLAs and has been used for the quality control and discrimination of Dendrobium recorded in Chinese pharmacopeia [22]. In this study, we treated 3 × Tg-AD mice with DDB and DNLAs for seven consecutive months from the pre-symptomatic stage of AD (five months of age), and these mice were then used to investigate the preventive effects of DDB and DNLAs on cognition and memory at twelve months of age. The present results revealed that 3 × Tg-AD mice pretreated with DDB and DNLAs could effectively attenuate cognitive and memory deficits. DDB and DNLAs also ameliorate amyloidogenic APP processing and tau hyperphosphorylation and eventually prevent hippocampal dendritic spine degeneration of 3 × Tg-AD mice, as well as up-regulate synaptic protein expression. This study demonstrates that DDB is the major active alkaloid compound of DNLAs, exerting a neuroprotective effect against AD.

We confirmed that DDB and DNLAs improved cognitive and memory deficits in twelve-month-old 3 × Tg-AD mice through a series of behavioral tests. Nesting behavior is one of many innate behaviors that are critical for the animal’s self-preservation and survival [23]. Abnormal nesting behavior was observed in 3 × Tg-AD mice at twelve months of age. In addition, these mice showed significant work memory deficits in a spatial object location task and the Morris water maze test, whereas the memory impairment and cognitive dysfunction in the 3 × Tg-AD mice were remarkably alleviated within seven months of continuous DDB administration, consistent with alleviating the effects of DNLAs on cognitive and behavioral impairment in 3 × Tg-AD mice (Figure 1 and Figure 2), in SAMP8 mice [13,24], in APP/PS1 mice [11], and in lipopolysaccharide- and Aβ-induced AD animal models [10,15]. However, the motor function and locomotor activity did not display significant differences between different groups in the rotarod and open field test.

It has been proved that AD is a disease of protein misfolding and abnormal accumulation, which may be relevant to cognitive deficits [25,26]. Autopsies and neuroimaging studies in AD patients indicate senile plaques, neurofibrillary tangles, and a large loss of brain neurons, especially in the hippocampus [27]. The mammalian hippocampus is associated with many crucial brain functions such as learning, memory, and adult neurogenesis [28]. Senile plaques are complicated lesions composed of a variety of Aβ peptides. Aβ is derived by proteolysis of the APP via BACE1 and PSEN1, released and deposited into extracellular senile plaques [29,30]. Tau pathology is another critical pathological cause of AD, and hyperphosphorylated tau contributes to learning and memory deficits in AD [31]. According to previous studies, DNLAs are not only effective in attenuating amyloid pathogenesis by decreasing APP and Aβ but can also attenuate tau pathology [14]. In this study, we observed that in addition to imitating the abnormal behavior of AD patients, 3 × Tg-AD mice also showed typical pathological changes, including the Aβ deposition and hyperphosphorylation of tau at twelve months old. DDB administration effectively inhibits the amyloidogenic processing of APP, resulting in the reduction in APP, sAPPβ, CTFβ, and Aβ. Given the little influence in the levels of BACE1 and PSEN1 with DDB treatment, we speculate that APP was degraded in a secretase-independent manner. The mechanisms underlying this effect require further study. We also demonstrated that treatment with DDB exerted a reversed effect on the phosphorylation of tau protein at sites Ser202/Thr205, Ser396, and Ser404 in the hippocampus of 3 × Tg-AD mice. Phosphorylation of tau protein and AD pathogenesis is modulated by several kinases, especially GSK3β [32]. Phosphorylation of GSK3β at the Ser9 site is a primary mechanism that inhibits GSK3β activity [33]. As shown in our data, DDB treatment for seven months repressed GSK3β activation by inhibiting the Ser9-GSK3β protein expression, thus ameliorating tau hyperphosphorylation.

Synaptic dysfunction is an early event in AD development, which underlies cognitive and memory deficits in AD patients and animal models [34,35]. Meanwhile, in the AD brain, both excessive Aβ and hyperphosphorylation of tau are destructive to synapses [36]. These structural and functional damages of synapses are closely related to cognitive dysfunction in AD patients [37,38]. Thus, DDB-induced attenuation in both APP and tau pathology might also contribute to the prevention of neuronal loss and synaptic degeneration and improved cognitive impairment in 3 × Tg-AD mice. In the present study, DDB administration successfully preserved dendritic spine density in the hippocampus of twelve-month-old 3 × Tg-AD mice, consistent with our previous findings showing that DNLAs enhanced neuronal survival and synaptic repair in rat primary cultured hippocampal neurons [39,40,41].

The expression of functional synaptic proteins is required for the growth of dendrites and synaptic connections during the development of the nervous system, so as to accurately control synaptic formation [18]. Synaptophysin is a synaptic vesicle marker that regulates neurotransmission and secretory vesicle recycling, and is closely linked to synaptic plasticity [42]. NCAM is enriched in neuronal synapses and modulates synaptic development, including early synaptic formation and strength [43]. The post-synaptic scaffolding protein PSD95 is an essential regulatory factor in axo-dendritic contact establishment [44]. We also investigated the preventive effects of DDB exposure on synaptic protein expression levels in twelve-month-old 3 × Tg-AD mice and found that it decreased the level of synaptophysin, PSD95, and NCAM in the hippocampus. However, these synaptic protein deficiencies were rescued by DDB treatment, which showed significant increases in synaptic protein PSD95, synaptophysin, and NCAM.

DNLAs have been demonstrated not only to induce autophagic and lysosomal acidification in neurons [11] but also to suppress inflammation both in vivo [14] and in vitro [16]. Lysosomal dysfunction has been involved in the pathogenesis of neurodegenerative diseases, including Alzheimer’s disease [45,46]. Improving autolysosomal acidification has been implicated as a key driving factor in suppressing neuronal impairments through attenuating neuroinflammation and neurotoxicity in reactive astrocytes [47,48,49]. Notably, recent studies have revealed that DDB could improve mitochondrial function, antioxidant and inhibiting inflammation [50,51]. Further investigating the role of DDB, like DNLAs, on the autophagy process and neuroinflammation would help clarify potential therapeutic targets for AD.

## 5. Conclusions

The neuroprotection of DNLAs extract DDB may be relevant to attenuated AD-like cognitive failures, decreased neural and synaptic damage, promoted APP and Aβ clearance, and alleviated tau pathologies in 3 × Tg-AD mice. However, the neuroprotective effect of other monomer components extracted from *Dendrobium nobile* requires further elaboration.

## Data Availability

The data are not publicly available due to applying for a patent. The data presented in this study are available on request from the corresponding author.
